# Effect of Cytokine Signaling 3 Gene Polymorphisms in Childhood Obesity

**DOI:** 10.4274/jcrpe.3167

**Published:** 2016-12-01

**Authors:** Mehmet Boyraz, Ediz Yeşilkaya, Fatih Ezgü, Aysun Bideci, Haldun Doğan, Korkut Ulucan, Peyami Cinaz

**Affiliations:** 1 Gazi University Faculty of Medicine, Department of Pediatric Endocrinology, Ankara, Turkey; 2 Private Doctor; 3 Intergen Genetics Center, Ankara, Turkey; 4 Marmara University Faculty of Dentistry, Department of Medical Biology and Genetics, İstanbul, Turkey; 5 Üsküdar University Faculty of Engineering and Natural Sciences, Department of Molecular Biology and Genetics, İstanbul, Turkey

**Keywords:** Cytokine signaling, polymorphism, obesity, children

## Abstract

**Objective:**

Although polymorphisms in suppressor of cytokine signaling 3 (SOCS3) was reported to be related to obesity, Metabolic syndrome (MS), and type 2 diabetes mellitus in various adult studies, there is a lack of data in children. In this study, we examined eight reported polymorphisms of SOCS3 in obese Turkish children and adolescent with and without MS and compared the results with that of controls.

**Methods:**

One hundred and forty eight obese and 63 age- and sex-matched control subjects were enrolled in the study. Obesity classification was carried out according to body mass index. World Health Organization and National Cholesterol Education Program criteria were used for the diagnosis of MS. Genotyping procedure was carried out by polymerase chain reaction and Sanger sequencing protocol.

**Results:**

The frequency of rs2280148 polymorphism was significantly higher in obese subjects with MS than in the control group, whereas the frequency of rs8064821 polymorphism was significantly higher in obese subjects with MS than in obese children without MS.

**Conclusion:**

The significant associations of certain SOCS3 polymorphisms with obesity parameters in both MS and MS -related insulin resistance, hypertension, and fatty liver suggest that polymorphisms in this gene may play a role in the pathogenesis of MS and also that they can be potentially used as a marker for attenuated or aggressive disease.

**WHAT IS ALREADY KNOWN ON THIS TOPIC?**

There is a relation of Metabolic syndrome (MS) with leptin and insulin levels.

**WHAT THIS STUDY ADDS?**

Suppressor of cytokine signaling polymorphisms also affect MS, obesity, and morbid obesity.

## INTRODUCTION

Obesity [Online Mendelian Inheritance in Man® (OMIM) #601665] is a multifactorial disease arising from the interaction between genetic and environmental factors (1). The incidence of obesity in childhood has been increased significantly during the recent years. Possible role of genetic and epigenetic factors involved in the pathophysiology of obesity are not fully known yet. Especially the relation of these factors with the severity of the disease in childhood has rarely been studied. It was previously shown that childhood obesity leads to atherosclerosis in later life, which starts as a chronic inflammatory process related to elevated cytokine levels (2).

Cytokine functions are regulated by suppressor of cytokine signaling proteins (SOCS). SOCS proteins are negative regulators of the JAK/STAT pathway and inhibit cytokine signaling (3). It is considered that SOCS proteins can attenuate signaling by inhibiting JAK activity or by promoting protein degradation (3,4). The human SOCS3 maps to chromosome 17q25.3 and consists of two exons spanning 2.729 nucleotides. The coding sequence in exon 2 (total of 2.401 nucleotides) comprises 678 nucleotides. Some of the single nucleotide polymorphisms (SNPs) on SOCS3 were previously shown to be related to obesity and type 2 diabetes mellitus in adults although the exact functional mechanisms are unclear (5,6,7,8). It has been reported that excessive secretion of SOCS3 may cause insulin resistance and play a role in hepatic fatty acid synthesis (8,9). The association between previously reported SNPs on SOCS3 and the parameters of childhood obesity with or without Metabolic syndrome (MS) is not well-known.

The aim of the present study is to determine the possible relations and prevalence of eight SNPs (-1044 C>A, rs12059, rs1061489, rs17849241, rs2280148, rs8064821, rs12953258, and rs4969169) defined previously on SOCS3 gene in obese and extremely obese children with or without MS.

## METHODS

### The Selection of Obese and Control Children and Adolescents

In the current study, 148 (66 female, 82 male; age range, 8 to 16.4 years) children and adolescents followed in Gazi University Faculty of Medicine, Department of Pediatrics, Division of Pediatric Endocrinology Clinic with a diagnosis of obesity were enrolled. Sixty-three (34 female, 29 male; age range, 8.5 to 18 years) sex- and age-matched healthy children and adolescents were enrolled as the control group. Volunteer subjects in both groups who met the inclusion criteria were selected randomly. Approval for the study was obtained from the Ethics Committee of Gazi University Faculty of Medicine, and informed consent was obtained from parents. Obese subjects only with diabetes, hypertension, hyperlipidemia, hypothyroidism, Cushing’s syndrome, severe chronic disease, acute illness, as well as genetic and metabolic diseases and syndromes were excluded from the study. The control group consisted of non-obese subjects without any chronic disease or infection.

### Anthropometric and Biochemical Evaluations

All anthropometric measurements were performed in the morning with underclothes and without shoes. Body height and weight were measured twice, and the mean values were recorded. Body height was measured with a Harpenden stadiometer and approximated to the nearest 0.1 cm. Individuals were weighed twice using a portable digital scale, and these values were also approximated to the nearest 0.1 kg; remeasurement was performed if the first two measurements differed by >0.2 kg. Body mass index (BMI) was calculated by dividing weight in (kilograms) by height in meters squared. Children with a BMI at or above the 95 percentile for their age and gender were classified as obese (10). A BMI of 40 or above was considered to indicate severe obesity (morbid obesity) (11). By a tape measure, waist circumference was measured from belly pit with loose belly and hip circumference was measured around the great trochanters while the patient is upright. Waist/hip ratio was calculated and recorded. The method used in measuring the waist circumference and the percentile values were obtained from the study of Hatipoglu et al (12). Blood pressure was measured while the patient was seated after a 30-minute rest. Blood pressure levels above the 95th percentile for age, gender, and height was defined as hypertension (13). Detailed family histories were obtained and physical examinations were performed. World Health Organization and National Cholesterol Education Program criteria for the diagnosis of MS were utilized in this study (14,15). Venous blood samples were collected into tubes containing no anticoagulant. The tubes were centrifuged (4000 rpm) at room temperature for 10 min to separate the serum, and the serum samples were stored at -80 0C until analysis. Leptin, insulin, fasting plasma glucose (FPG), total cholesterol (TC), high-density lipoprotein cholesterol (HDL-C), and triglyceride (TG) concentrations were measured. Serum insulin, TC, HDL-C, TG, and FPG measurements were performed by using the Abbott-Aeroset autoanalyzer (Chicago, IL, USA) with original kits. Low-density lipoprotein cholesterol (LDL-C) levels were calculated using the Friedewald equation. Serum leptin levels were determined by using an enzyme-linked immunosorbent assay (ELISA) kit (DRG International Inc., NJ, USA). The inter-assay and intra-assay coefficients of variation for this method were 6.6% and 4.6%, respectively. Homeostasis model assessment for insulin resistance (HOMA-IR) was used to estimate the insulin resistance in our population (15,16). The HOMA-IR index was calculated using the following formula, [FPG (mmol/L)×fasting serum insulin (mU/mL)]/22.5. Obese patients in the study group were divided into two groups as insulin resistant [IR (+)] if their HOMA-IR values were above 3.16 and non-insulin resistant [IR (-)] if their HOMA-IR values were lower than 3.16 (15).

Liver steatosis was evaluated using ultrasonographic examination and classified according to the criteria defined by Saverymuttu et al (17). Ultrasonographic examination of the liver was performed by an experienced radiologist, using a high-resolution B-mode ultrasonography system (General Electric LOGIQ 500, convex 3-5 MHz). The radiologist was masked to all clinical and biochemical characteristics of subjects.

### Genotyping

Genomic DNA was extracted from peripheral blood using NucleoSpinR Blood kit (MN GmbH, Germany), according to the manufacturer’s protocol. Four polymerase chain reaction (PCR) primer sets were designed for the amplification of SNP regions. For PCR amplification, SuperHoTTaq (Bioron GmbH, Germany) was used as Taq polymerase and the reaction was carried out with an annealing temperature of 60 0C. The final concentrations of reagents in PCR were as follows: 1.5 mM of MgCl2, 0.2 UM of each primer, and 0.2 mM of each dNTP. A duplex PCR was performed (two tubes for each sample), each with a volume of 25 U. After the confirmation of PCR by agarose gel electrophoresis, two duplex PCR tubes were combined to get ready for genotyping. Genotyping of the 8 SNPs in all 211 samples was carried out by using SNaPshotR Multiplex Kit (Applied Biosystems Inc, USA), according to manufacturer’s protocol. SNPs were confirmed by direct DNA sequencing. Primer sets used in this study can be sent upon request.

### Statistical Analysis

Statistical Package for the Social Sciences for Windows v.11.5 (Chicago, IL, USA) was used in the statistical analysis. Genotype and allele frequencies in SOCS3 polymorphism were compared separately between groups. Descriptive statistics are given as mean ± standard deviation, frequency, and percentage. The groups were compared using the t-test or Mann-Whitney U test, as appropriate. Logistic regression analysis was used to calculate the odds ratios and 95% confidence interval values. A p-value of <0.05 was considered statistically significant.

## RESULTS

Totally eight SNPs (-1044 C>A, rs12059, rs1061489, rs17849241, rs2280148, rs8064821, rs12953258, and rs4969169) in SOCS3 were examined. Four of the examined SNPs (-1044 C>A, rs12059, rs1061489, and rs17849241) were not detected in any of the individuals.

### Obese Group vs. Control Group

Biochemical and anthropometric features of the groups are given in Table 1. Except TC and LDL-C levels, significant difference was found in other parameters between control and obese groups (p<0.05). For the genotype and allele frequencies, no significant difference was found between control and obese groups (p>0.05) (Table 2).

### Control Group vs. Morbid Obese Group

Except TC and LDL-C levels, significant difference was found between control and morbid obese groups (p<0.05). For the genotype and allele frequencies, no significant difference was found between control and morbid-obese groups (p>0.05) (Table 3).

### Morbid Obese vs. Non-Morbid Obese Groups

Except waist circumference, systolic blood pressure, TC, HDL-C, LDL-C, and TG levels, significant difference was found between morbid obese and non-morbid obese group for the other parameters studied (p<0.05). Although genotype frequencies of polymorphisms in subjects with morbid obesity were not different from non-morbidly obese patients, A-allele carrier frequency was significantly higher than C allele carrier frequency at rs2280148 SNP locus in morbidly obese subjects (Table 4).

### Control Group vs. Obese Patients with Metabolic Syndrome

Of the148 obese patients, 16 children (10.8%) had MS. Except TC and LDL-C levels, significant difference was found between controls and obese patients with MS (p<0.05). The AC genotype frequency was higher in obese patients with MS than control group at rs2280148 SNP locus (p<0.05). On the contrary, AA genotype frequency at the same locus was significantly higher in control group than obese children with MS (p<0.05) (Table 5).

### Obese Children with Metabolic Syndrome vs. Those without Metabolic Syndrome

Although BMI, waist circumference, blood pressure, fasting insulin, HOMA-IR value, and TG levels of children with MS were significantly higher than the values of those without MS, HDL-C levels were found to be significantly lower (p<0.05). No significant differences were found between the two groups for FPG, leptin, LDL-C, and TC levels (Table 6). The GG genotype frequency at rs8064821 locus was significantly higher in non- MS obese group than MS obese group (Table 7).

### The Association of Genotypes with Risk Factors of Obesity

Insulin resistance was observed in 78 (52.8%) obese patients. There was no significant difference between insulin resistant, non-insulin resistant obese, and control groups according to genotype and allele frequency (data not shown). However, mean insulin levels in obese cases carrying the GT genotype were significantly higher than those of individuals having the GG genotype at rs8064821 SNP locus (p<0.01).

Ultrasonographic examination revealed moderate or severe fatty liver in 58 (39%) of obese subjects. The GG genotype frequency at rs8064821 locus of “non-fatty liver” group was significantly higher than “fatty liver” group among obese group. In addition, GT genotype frequency in obese subjects with fatty liver was significantly higher (p<0.05) (data not shown).

## DISCUSSION

SOCS is a family of intracellular proteins that negatively regulates cytokine signaling by interacting with cytokine receptors and signaling proteins. SOCS genes, especially SOCS3, display tissue-specific function and are expressed in many tissues and immune regulator cells. Of these, SOCS1 and SOCS3, expressed in beta cells, regulate the IFN-γ signaling pathway (18). These findings suggest that the SOCS molecules are implicated in the development of autoimmunity or allergy in human diseases (19).

In obese German children, two SNPs (-1044 C>A, rs12953258) in SOCS3 were analyzed and no difference was reported (9). Four (-1044 C>A, rs12059, rs1061489, rs17849241) of the eight polymorphisms were not detect in our population. We did not detect a significant difference between obese and control groups in terms of genotype and allele distribution of rs12953258 polymorphism and this was in agreement with the previous findings of Hölter et al (9). However, allele frequencies between morbidly obese and obese subjects were different. Accordingly, the frequency of A allele carrier at rs2280148 SNP locus in morbidly obese was significantly higher than C allele carrier frequency compared to obese group. This finding suggests that A allele carrier status at rs2280148 SNP locus may be a risk factor or a marker for morbid obesity. In recent years, it has been shown that the increase in the prevalence of obesity, may also lead to an increase in the prevalence of MS (20). SOCS3 polymorphisms may play a role in the development of MS components. In particular, SOCS proteins, by affecting insulin and cytokine signaling, play an important role in the pathogenesis of MS (21). However, there is not sufficient data to comment about the functional relation. Sixteen of our cases (10.8%) had MS. Although the frequency of AC genotype at rs2280148 SNP locus was higher in children with MS than in the control group, the frequency of AA genotype was significantly lower. This suggests that the presence of AC genotype at rs2280148 SNP locus may be a risk factor for MS, while the AA genotype seems to be a marker of uncomplicated obesity. In addition, the frequency of GG genotype at rs8064821 locus in obese patients without MS was found to be significantly higher than that in obese subjects with MS. Again, the presence of GG genotype at this locus is associated with an uncomplicated disease.

Increased cytokine levels in obese patients are suggested to be associated with insulin resistance seen in these patients. In the recent years, it has been proposed that SOCS proteins, especially SOCS3, have been extensively studied in the context of the regulation of insulin signaling (22). At least three mechanisms used by SOCS, leading to the inhibition of insulin signaling, have been revealed: 1- competition for binding to the activated insulin receptor (IR), 2- degradation of IR substrate (IRS) proteins, and 3- inhibition of IR tyrosine kinase activity (23). Decreased glucose levels have been detected in SOCS1 knockout mice, and cells derived from these mice exhibited increased insulin signaling (8). In another study, increased SOCS1 and SOCS3 were determined in insulin resistant obese animals (21). Three SNPs (rs4969169, rs12953258, and rs8064821) were studied in 2777 normal white twin women in the UK and no direct relation was detected between insulin sensitivity measures and leptin and serum lipids. The authors stated that SOCS3 polymorphisms alone do not play a fundamental role in the regulation of body weight in adults (24). Our results also do not support a major role for SOCS3 variants in body weight regulation in our female population. In a Danish study on 360 healthy young adults, rs12953258 polymorphism in SOCS3 was associated with insulin sensitivity (9). In the present study, the insulin sensitivity index in AA individuals was significantly higher than that in heterozygous carriers. In our study, there was no significant difference between obese with (78 cases) or without insulin resistance groups in terms of examined polymorphisms. However, there was a significant difference between subjects with GG and GT genotypes at rs8064821 polymorphism locus regarding insulin levels. Accordingly, the average insulin levels in the GT genotype carriers were higher than that of the GG genotype carriers. These results suggest that the presence of GT genotype in rs8064821 polymorphism locus may be one of the insulin-related factors in our society, and it is important to follow up the children with the related genotype till adulthood because development of insulin resistance is an ongoing process. In addition, similar to the study on twin women (24), we did not detect any relation between SOCS3 polymorphisms and leptin, TG, and TC levels.

Fatty liver is commonly seen in obesity. Ueki et al (21) demonstrated that over-expression of cytokine signaling suppressors in mice liver (especially, SOCS1and SOCS3) might increase the levels of sterol regulatory element-binding transcription factor 1c (SREBP-1c) protein which has a key role in fatty acid synthesis in the liver. SOCS1 and SOCS3 inhibition in the latter study increased insulin sensitivity, decreased SREBP-1c to normal levels, and ameliorated fatty liver and hypertriglyceridemia dramatically in obese diabetic mice. Therefore, reducing the expression of SOCS proteins in the liver may be useful in diabetes, in obesity-related MS, and in the treatment and prevention of fatty liver (21). Fatty liver was seen in 58 obese individuals in our study. The frequency of GG genotype at rs8064821 SNP locus was significantly higher in subjects without steatosis than in those with steatosis. The frequency of GT genotype was significantly higher in obese patients with fatty liver. These results suggest that, the presence of GT genotype may be a risk factor; conversely, presence of GG genotype shows a decreased probability of liver involvement. The presence of GG genotype at rs8064821 SNP locus was significantly higher in obese patients without hypertension, and the presence of GT genotype was significantly higher in hypertensive obese patients. Similarly, the presence of GT genotype may be a risk factor, while the presence of GG genotype shows a decreased risk for hypertension in obese patients. As far as we know, this is the only data available in children. For the prevention, early diagnosis, and treatment of metabolic complications, waist circumference measurement is recommended in children with central obesity (25). In obese, there is a significant relation of waist circumference with leptin, insulin, HOMA-IR, and BMI, however, no significant relation has been detected between blood lipids and FPG levels. No significant relation was detected between waist circumference values and SOCS3 polymorphisms and this was also not previously investigated. Like insulin, leptin is a key hormone involved in the regulation of energy balance and glucose homeostasis. Development of resistance to the action of this hormone which can occur with age, obesity, and inflammation appears to have a primary role in the pathogenesis of obesity and type 2 diabetes mellitus. SOCS family of proteins is now thought to have a role in the development of leptin resistance owing to their ability to inhibit leptin signaling pathway (26). Leptin signaling cascade may be the possible underlying mechanism of leptin resistance in obese patients. In fact, we detected a significant increase in leptin levels of the obese patients compared to that of the control group; conversely, we did not determine any significant difference between leptin levels and SOCS3 polymorphisms.

In conclusion, some SNPs in SOCS3 might be an important marker of attenuated or aggravated disease in childhood and adolescent obesity. To our knowledge, this is the first study that investigates the possible associations between the presence of SOCS3 polymorphisms and several risk factors for obesity. SOCS3 and in particular the high incidence of the variances of genotype rs8064821 and rs2280148SNPs in obese patients with MS may be important markers of insulin resistance, hypertension, or fatty liver, and more aggressive treatment of obesity may be considered in the presence of these polymorphisms. One of the weak points of our study is the number of the cases, which we think is not sufficient to make a proposal. It is obvious that more population-based genetic studies are needed to reveal the accuracy of this finding.

## Ethics

Ethics Committee Approval: Gazi University Ethics Committee of Medicine Faculty, 2008, Informed Consent: It was taken.

Peer-review: Externally peer-reviewed.

## Figures and Tables

**Table 1:**
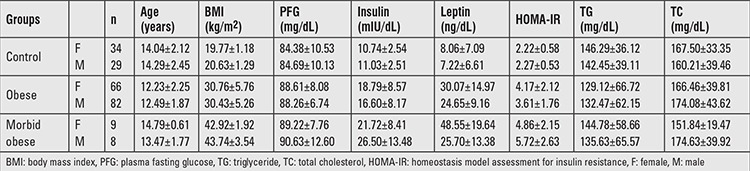
Features of obese, morbid obese, and control groups

**Table 2:**
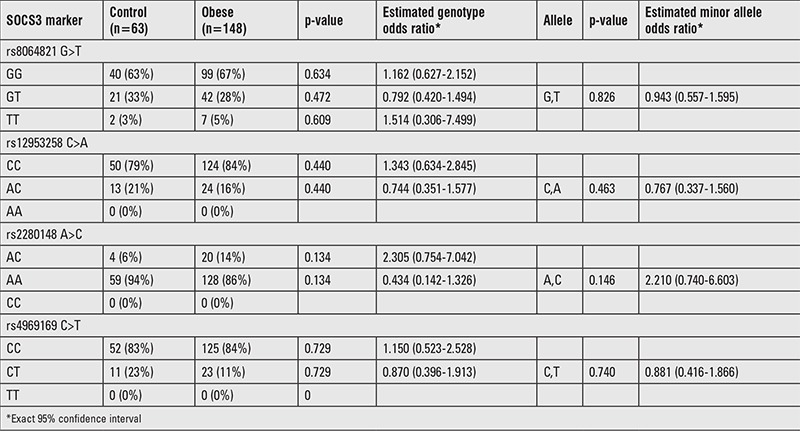
Genotype distributions and estimates of the polymorphisms in control and obese groups

**Table 3:**
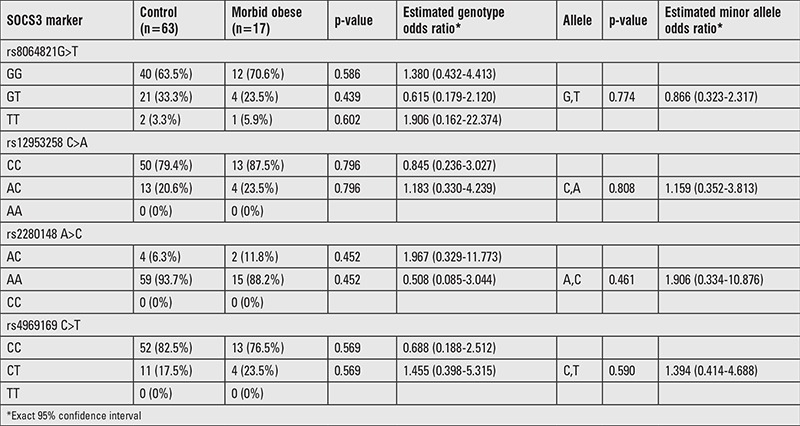
Genotype distributions and estimates of the polymorphisms in control and morbid obese groups

**Table 4:**
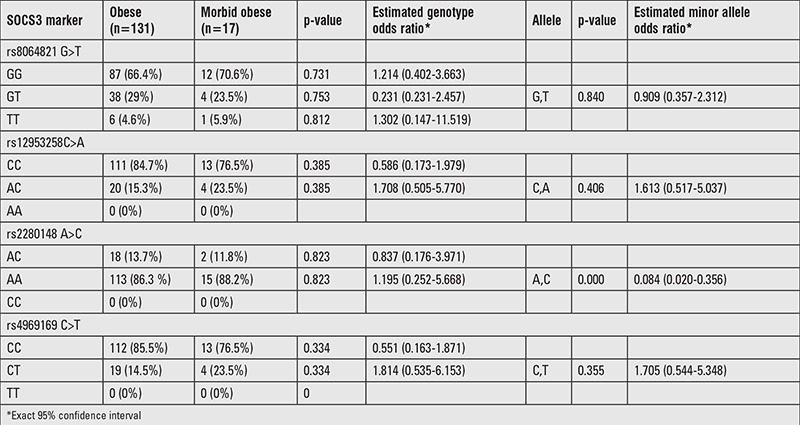
Genotype distributions and estimates of the polymorphisms in obese and morbid obese groups

**Table 5:**
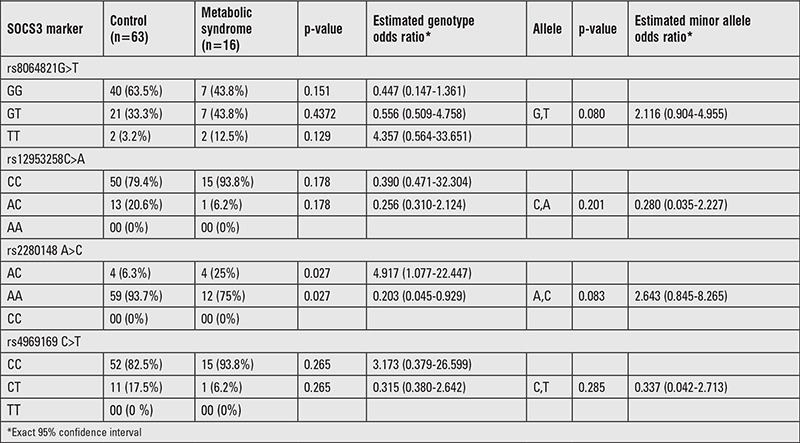
Genotype distributions and estimates for the polymorphisms of cases between control and metabolic syndrome

**Table 6:**
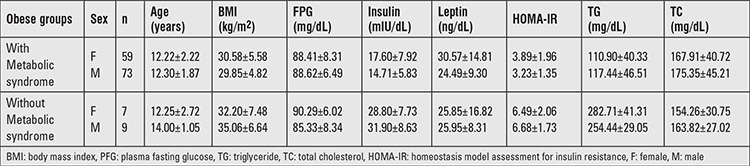
Features of obese children with metabolic syndrome and without metabolic syndrome

**Table 7:**
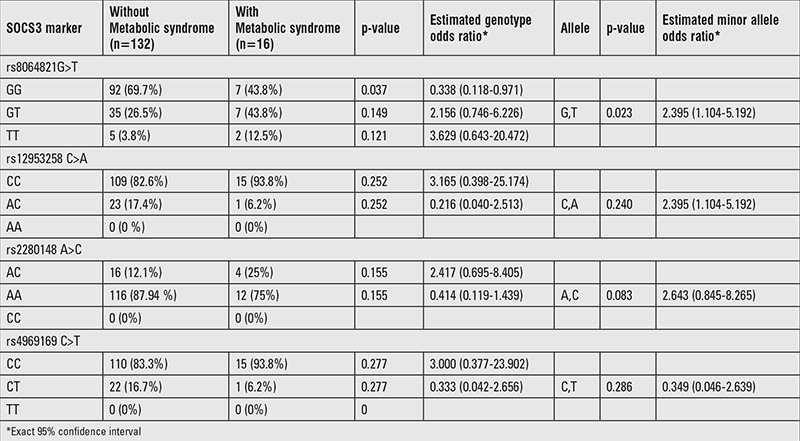
Genotype distributions and estimates for the polymorphisms of cases between with and without metabolic syndrome
